# Overexpression of Tau Downregulated the mRNA Levels of Kv Channels and Improved Proliferation in N2A Cells

**DOI:** 10.1371/journal.pone.0116628

**Published:** 2015-01-15

**Authors:** Xiantao Li, Ximu Hu, Xiaoqing Li, Xuran Hao

**Affiliations:** 1 Department of Neuroscience, College of Life Sciences, South-Central University for Nationalities, Wuhan, 430074, China; 2 South-Central University for Nationalities, Wuhan, 430074, China; McGill University Department of Neurology and Neurosurgery, CANADA

## Abstract

Microtubule binding protein tau has a crucial function in promoting the assembly and stabilization of microtubule. Besides tuning the action potentials, voltage-gated K^+^ channels (Kv) are important for cell proliferation and appear to play a role in the development of cancer. However, little is known about the possible interaction of tau with Kv channels in various tissues. In the present study, tau plasmids were transiently transfected into mouse neuroblastoma N2A cells to explore the possible linkages between tau and Kv channels. This treatment led to a downregulation of mRNA levels of several Kv channels, including Kv2.1, Kv3.1, Kv4.1, Kv9.2, and KCNH4, but no significant alteration was observed for Kv5.1 and KCNQ4. Furthermore, the macroscopic currents through Kv channels were reduced by 36.5% at +60 mV in tau-tranfected N2A cells. The proliferation rates of N2A cells were also improved by the induction of tau expression and the incubation of TEA (tetraethylammonium) for 48 h by 120.9% and 149.3%, respectively. Following the cotransfection with tau in HEK293 cells, the mRNA levels and corresponding currents of Kv2.1 were significantly declined compared with single Kv2.1 transfection. Our data indicated that overexpression of tau declined the mRNA levels of Kv channels and related currents. The effects of tau overexpression on Kv channels provided an alternative explanation for low sensitivity to anti-cancer chemicals in some specific cancer tissues.

## Introduction

It is well known that the microtubule-associated protein tau is expressed in the central and peripheral nervous system. However, tau is also found in many other tissues such as kidney, lung [1], muscle [2], and breast [3]. Tau is able to bind to both the outer and inner surfaces of microtubules, and plays an important role in promoting tubulin polymerization and stabilizing microtubules [4]. Tau has been divided into four regions: an amino-terminal projection region, a proline-rich domain, a microtubule-binding domain (MTB), and an acidic carboxyl-terminal region [5]. In adult human brain, six developmentally modulated isoforms are generated by alternative splicing around the amino-terminal region and MTB [6, 7]. The tau isoforms are distinct from each other by containing the different numbers of microtubule binding repeat sequences (R) and amino-terminal exons (N) [8].

Numerous studies have indicated that abnormally hyperphosphorylated tau was involved in the formation of neurofibrillary tangles (NFTs), a pathological hallmark of Alzheimer’s disease (AD) [9, 10]. Many serine and threonine residues on tau are phosphorylated by various kinases, which include glycogen synthase kinase 3 (GSK3), cyclic-AMP-dependent kinase (PKA), and microtubule-affinity regulating kinase (MARK) [11]. Dephosphorylation mediated by protein phosphatases counterbalances the effects of protein kinases; therefore, an imbalance of between protein kinases and protein phosphatases was considered as underlying mechanism of tau hyperphosphorylation in AD. The abnormal alterations of tau were also observed in many neurodegenerative disorders, and thus those diseases were referred to as tauopathies [11].

Previous works have shown that estrogen was involved in improvement of neuronal survival and cognitive function [12, 13]. Ferreira et al reported that estrogen-enhanced neurite growth was mediated by promoting the levels of tau protein in dissociated cultures of hypothalamic neurons [14]. In agreement with such study, overexpression of tau was able to promote neurite extension in cell culture [15]. Moreover, tau was able to obstruct the binding of taxane [3] and paclitaxel [16] to the inner surface of microtubules, and thus reduced the sensitivity of breast cancer tissues with expression of estrogen receptors (ER) to these drugs, showing a promoting role of tau in tumor growth. These results implied that tau was capable to improve cell growth under some pathophysiological conditions.

Ion channels have an essential role in cell proliferation and the development of cancer. On the other hand, cancer cells are, on average, more depolarized than healthy cells at the same histological origin [17, 18]. Kv channels, which contained six transmembrane segments and one pore-forming region, show a wide range of voltage dependence and kinetic properties. These channels are widely expressed in different kinds of excitable and non-excitable tissues, and have a crucial role in tuning action potential and neuronal excitability [19]. Accumulated data suggested that Kv channels also were considered as an important modulator in the process of cell growth [20]. The treatment of alpha-DTX inhibited Kv 1.1 currents in MCF-7 cells, and improved the proliferation rate of these cells [21]. In germinal tumours, an increase of Kv7.1 (KCNQ1) and KCNE1 subunits has been found [22]. Although there have been extensive research efforts, the exact mechanisms underlying the involvement of Kv channels in the pathogenesis of cancers remained unclear.

As mentioned above, enhanced expression of tau by estrogen stimulation is less sensitive to taxane chemotherapy in human breast cancer cell lines [3], suggesting that tau may play a promoting role in cell proliferation. Although Kv channels were involved in the development of tumors, little is known about the relationship between tau and Kv channels in the pathogenesis of tumors.

The mouse neuroblastoma N2A cells functionally expressed Kv channels [23] and tau protein [24, 25], and were frequently taken as a model by many groups to perform the related studies. In the experiments here, we also took N2A cells as a model to investigate the interaction of tau with Kv channels. Quantitative Real Time PCR (QPCR) was used to measure the change of expression of Kv channels after tau transfection, and whole-cell recordings were conducted to detect the alteration of related Kv currents. The proliferation rates of N2A cells after treatment with tau transfection and Kv channel blocker were assessed further. To define the direct correlation between tau and Kv channels, related plasmids were transfected into HEK293 cells to assess the possible interaction.

## Materials and Methods

### Cell culture and plasmid transfection

Mouse neuroblastoma N2A or HEK293 cells were grown in Dulbecco’s modified Eagle’s medium (DMEM), supplemented with 10% fetal calf serum, 100 U/ml penicillin and 100 ug/ml streptomycin at 37°C in 95% humidified air with 5% CO_2_, as previously described [26]. Tau (human tau40, 441 residues) subcloned into pIRES2-EGFP vector (Clontech) by ligating into the EcoRI/BamHI sites was kind gifts of Dr. J. Z. Wang (Huazhong University of Science and Technology, China). Kv2.1 subcloned into pEYFP-N1 vector (Clontech) by ligating into the XhoI/EcoRI sites was generously provided by Dr. L. Kaczmarek (Yale University, the School of Medicine, New Haven, USA). These plasmids were transfected into N2A cells or HEK293 cells using Lipofectamine 2000 (Life Technologies, Bethesda, MD) according to the manufacturer’s protocol. Opti-MEM I Reduced Serum Medium without serum was employed as a medium for transfection.

### Western blotting

Tau-transfected N2A cells were harvested and then lysed in the sample buffer (50 mM, Tris pH 7.6; 2% sodium dodecyl sulfate; 10% glycerol; 0.2% bromophenol blue and 3 mM PMSF) for 30 min on ice. A low-speed centrifugation of 1,000 × g for 3–5 min at 4°C was conducted to remove nuclei and cell debris. Proteins were then separated by 10% SDS-PAGE and transferred to nitrocellulose (NC) membrane. After blocking for 1 h in a solution of 5% non-fat dry milk in TBS (tris buffered saline)/Tween 20, membranes were immunoblotted with antibody Tau-5 (Lab Vision; 1:1000 dilutions) against total tau or antibody DM1A (Sigma; 1:1000 dilutions) against α-tubulin. Horseradish peroxidase-conjugated mouse anti-goat IgG (Rockford; 1:3,000 dilutions) was added for 1 h at room temperature for visualization.

### Cell proliferation assay

Cell proliferation was measured by a WST-8 (2-(2-methoxy-4-nitrophenyl)-3- (4-nitrophenyl)-5-(2, 4-disulfophenyl)-2H-tetrazolium) assay (Beyotime) according to the manufacture’s protocol. After suspending N2A or HEK293 cells in DMEM medium without phenol red, the 100 µl of cell suspension (1000 cells/well) was plated in 96-well plates and grown for 24 h; then tau40 was transfected into cells using Lipofectamine 2000. In some cases, cells were treated with TEA, a general blocker of Kv channels. TEA was diluted in the medium prior to application to the cells. WST-8 assay reagents (10 µl) were added into medium after these cells were cultured for 48 h. Following, cells were incubated at 37°C for 1–4 h and the absorbance at 450 nm was detected by a microplate reader.

### PCR analysis

Total RNA was prepared from N2A or HEK293 cells using the TRIzol reagent (Invitrogen, Carlsbad, CA) and further treated with DNase I (Invitrogen). The concentration of total RNA was defined by measurement of the absorption at 260 nm. Total RNA (1 μg) was reverse transcribed (RT) into first strand cDNA with Oligo(dT) and M-MLV reverse transcriptase (Invitrogen).

The regular PCR was conducted for the analysis of Kv expression in N2A or HEK293 cells. The sense and antisense PCR oligonucleotide primers chosen to amplify cDNA were present in Table 1. BLAST searches were conducted to confirm primers had no homology with any other known gene products. One microliter of the first-strand cDNA reaction mixture (0.5 µg/µl) was used in a 25 µl PCR consisting of 0.4 nM of each primer, 10 mM Tris-HCl, 50 mM KCl, 1.5 mM MgCl_2_, 200 μM each dNTP, and 0.625 units of Taq DNA polymerase. The PCR was performed at following conditions: 95°C for 5 min as an initial melt, followed by 18–40 cycles of 95°C for 30 s, annealing at 60°C for 30 s, extension at 72°C for 30 s; followed by a final extension at 72°C for 10 min. PCR products were visualized on 1.5% agarose gels staining with 0.5 mg/ml ethidium bromide.

**Table 1:** Oligonucleotide sequences of primers used for RT-PCR.

**Gene**	**Accession numbers**	**Forward Primer**	**Reverse Primer**	**Length (bp)**
Kv1.1	NM_010595.3	AGGGCTCCCGTAGTGTTC	TTTGCTGCTCCTTGGCTC	457
Kv1.2	NM_008417.5	CTCCTCAAGTCGTGGTGC	GGTCTGCCTCTGGGTCAT	469
Kv1.3	NM_008418.2	GTGTCAGTGCTGGTCATTCTC	CTGCCCATTACCTTGTCGT	351
Kv1.4	NM_021275.4	TTCGGAGAACCTTGACTT	GACGCAGTTCCAGCAGAG	445
Kv1.5	NM_145983.2	GTCACCCATCAAAGTCCG	CACTCGTCAGCCATAAGAATA	348
Kv1.6	NM_013568.6	GTGGATGATGTAACCGTGTC	CTCCTTCTCCTCCTCTGG	454
Kv1.7	NM_010596.2	AGACACTGCCAGACTTCCG	AGGACCACGCCAATGAAG	490
Kv2.1	NM_008420.4	AGGAGCAGATGAACGAGG	AGTGACAGGGCAATGGTG	202
Kv2.2	NM_001098568.2	GCGACTGTAACACTCACG	AGCAATGGTGGAAAGAAC	434
Kv3.1	NM_001112739.2	TCGCTCACATCCTGAACTAT	CGTTCTCGATTTCGGTCT	489
Kv3.2	NM_001025581.1	TTGATATTCGCTACGATG	TTCTGGAGGTGATAATGG	489
Kv3.3	NM_001290682.1	GAGGCACTGGACTCTTTCG	CACCGTCTTGTTGCTGATGT	378
Kv3.4	NM_145922.2	GAATCGCCCATTTACTGC	GCCTTCTTGTTTCTGTCCC	251
Kv4.1	NM_008423.1	TGGCGACGGACAAATGCT	CCGGCTGTCACAGTTCAGGT	397
Kv4.2	NM_019697.3	TCTCAAGGGCTGCGTATA	TTCGTTTGTCTGCTCGTT	349
Kv4.3	NM_021275.4	CAGGAAACGGTAGGAATC	GGAGTTCAGGGATGAT	342
Kv5.1	NM_201531.3	TGCCTCCTCTTCACATTTC	CTGGGCTTGGTCTTCTATT	419
Kv6.1	NM_001081134.1	TTTGGCACCATCCTCACC	TGGCACATCTGGGAACACT	428
Kv6.2	NM_001190373	TGGAAACAGCCGAGAACAA	GCTGCGGTCGAAGAAGAA	440
Kv8.1	NM_0026200.3	CTCAGGCGTTCCGTTATG	GATGGACACTGCCACAAA	353
Kv9.1	NM_008435.2	ACCCGCTCAACCTCATTG	GTAAGATGCCCACCTCACG	240
Kv9.2	NM_001271704.1	CACAGGCAAGGTGTAGCG	TCAGCCATGACGTGAAGC	337
Kv9.3	NM_173417.3	GCTGTATGACATCGCCACC	CTCCTTGCGCTCCTGGTA	462
KCNH1	NM_010600.2	ACTTATCCGCATGAACTACCT	CTTCCCTGAACCAGACCC	463
KCNH2	NM_013569.2	ATTCCCAACCCATTACGC	TTTCCAGGACGGGCATAT	386
KCNH4	NM_001081194.2	GCAGAAGTTAGAGCGGTACTCA	CCGAGCCATTGACATAAGG	206
KCNQ1	NM_008434.2	ACTTCACCGTCTTCCTCATT	TGGCGAACACTTGTCCTT	294
KCNQ2	NM_010611.2	CTCAAGGTGGGCTTCGTG	GCAATGGAGGCAATCAGC	485
KCNQ3	NM_152923.2	CTGGGCTCGGCTATCTGT	GTGCTTCTGACGGTGCTG	354
KCNQ4	NM_001081142.1	TTGTCGCTACAGAGGATGGC	CAGGAAAGAGGCAAAGATGAG	295
GAPDH	NM_001289726.1	AGGCCGGTGCTGAGTATGTC	TGCCTGCTTCACCACCTTCT	530

Abbreviation: Kv, voltage-gated K^+^ channel; GAPDH, glyceraldehyde-3-phosphate dehydrogenase.

An Agilent Stratagene Mx3000P sequence detection system was taken to perform QPCR stained with SYBR I green dye. Each SYBR Green reaction (20 μl totals) contained 50 ng cDNA, 10 μl SYBR Green PCR Master Mix, 5 μl water, and 250 nM forward and reverse primers.

Samples were denatured at 95°C for 4 min, and then cycled 40 times at 95°C for 30 s; followed by annealing and extension at 60°C and 72°C for 30 s, respectively. An additional extension was conducted at 72°C for 10 min. A final dissociation step was run at 95°C for 1 min, 60°C for 30 s, and 95°C for 30 s. The sense and antisense oligonucleotide primers chosen for QPCR were showed in Table 2. The water was used as a non-template control to confirm the integrity of reaction components in all QPCR. Data for each sample were normalized to GAPDH and presented as relative mRNA values.

**Table 2:** Oligonucleotide sequences of primers used for QPCR.

**Gene**	**Accession numbers**	**Forward Primer**	**Reverse Primer**	**Length (bp)**
Kv2.1	NM_008420.4	GAGAAGCCCAACTCATCGGT	TGAGTGACAGGGCAATGGTG	88
Kv3.1	NM_001112739.2	CCCTACTCATCCCGCTAC	TCGATTTCGGTCTTGTT	128
Kv4.1	NM_008423.1	CATCCGCCTTGCCAACTCTA	GCTTGGCATTGAGGCTTGAG	119
Kv5.1	NM_201531.3	TGCCTCCTCTTCACATTTC	CTGGGCTTGGTCTTCTATT	419
Kv9.2	NM_001271704.1	GAAGGAGAAGACGCACAG	GCGATGACTACGATGACG	128
KCNH4	NM_001081194.2	CGGCACGCACAGCAACTT	CCATAGAGGAACCGACAGC	147
KCNQ4	NM_001081142.1	ATGTCATGCCTGCTGTGA	CCTTGAATTTCCTTTTGG	78
GAPDH	NM_001289726.1	CCAACAGAGGACGCTGAA	TCACTGCCACCCAGAAGA	316

Abbreviation: Kv, voltage-gated K^+^ channel; GAPDH, glyceraldehyde-3-phosphate dehydrogenase.

### Electrophysiology

Kv currents in N2A or HEK293 cells were recorded using the whole-cell patch clamp technique. EPC-9 patch-clamp system (HEKA Elektronik, Lambrecht, Germany) was used for data acquisition and analyses. The bath solution contained (in mM): 75 Na-gluconate, 70 NaCl, 5 KCl, 1 MgCl_2_, 5 HEPES, 5 glucose, with pH adjusted to 7.4 using NaOH; the pipette solution contained (in mM): 150 KCl, 2 MgCl_2_, 5 HEPES, 5 EGTA, 5 Glucose, 5 Na_2_ATP, with pH adjusted to 7.3 using KOH. N2A cells grown on coverslips were transferred to a chamber, which was mounted on the stage of an inverted microscope (Olympus IX 70). Patch pipettes were pulled from borosilicate glass capillaries. When immersed in standard extracellular solution, the pipette resistance was between 1.5 and 3.0 MΩ. All experiments were carried out at room temperature (22°C).

For the construction of current-voltage (I-V) relations, the amplitudes of currents at each test potential were measured as average amplitudes between start currents and end currents. The decay phases of the currents elicited during long (5 s) depolarizing voltage steps to test potentials from a holding potential of -80 mV were fitted by single exponential using the following expression: f(t) = A exp(-t /τ) + Ass, where t is time, τ is the time constants of decay of the inactivating Kv currents, A is the amplitudes of the inactivating current components, and Ass is the amplitude of the steady-state, noninactivating component of the total outward current. Time zero was set at the peak of the outward current for all fits.

### Analysis and statistics

Results are presented as mean ± SE. Statistical significance was calculated using paired Student’s t-test or one-way analysis of variance (ANOVA). A value of P < 0.05 was accepted for statistical difference.

## Results

### The mRNA expressions of Kv channels in N2A cells

**Figure 1:**
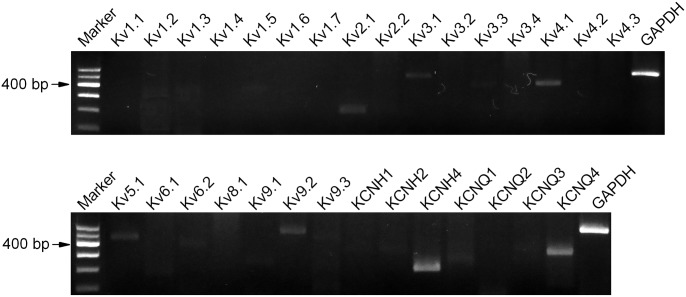
The mRNA expression of Kv channels. The PCR product of GAPDH (316 bp) at right lane was taken as positive control. N2A cells displayed bands for Kv2.1, Kv3.1, Kv4.1, Kv5.1, Kv9.2, KCNH4, and KCNQ4, corresponding to expected length shown in Table 1. The first lane showed the DNA maker.

Kv channels were widely expressed in a variety of neural cells, including axon and astrocyte. Besides tuning the action potential and neuronal excitability [19], Kv channels have been reported to be involved in axon development and growth [27]. To explore the possible linkages between tau and Kv channels, we first clarify the expression of these channels in N2A cells. Several specific primers to detect mRNA of targeted Kv channels were designed and present in Table 1. Positive signals for Kv channels were certainly detected in N2A cells by regular PCR (n = 8), including Kv2.1, Kv3.1, Kv4.1, Kv5.1, Kv9.2, KCNH4 and KCNQ4 (Fig. 1). Thus, these Kv channels were chosen for further study. The PCR product of GAPDH, a house-keeping gene, was also detected at the expected size (316 bp) and used to confirm RT (Fig. 1).

### Overexpression of tau reduced the mRNA levels of Kv channels and the macroscopic currents through these channels

**Figure 2:**
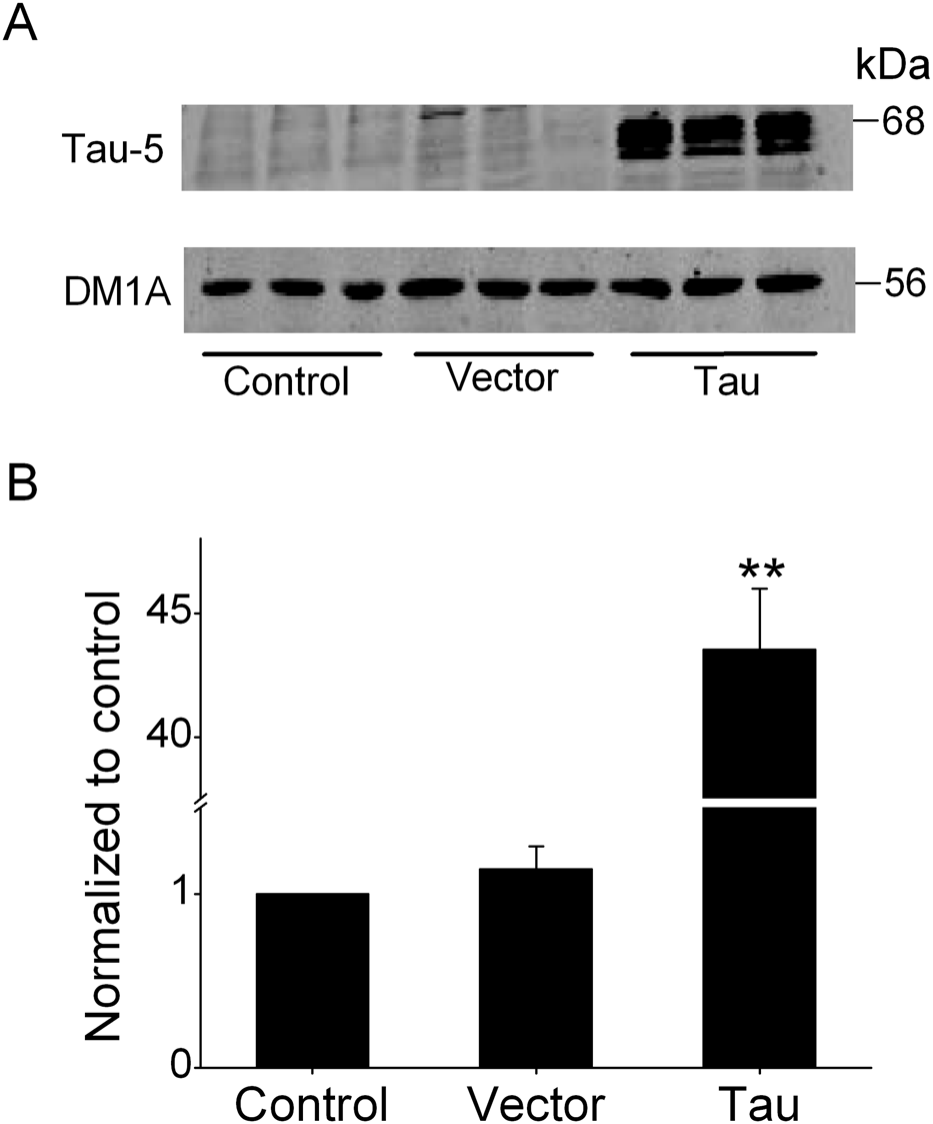
Analysis for tau overexpression after transfection. (A) Western blotting analysis showed that the expression of tau was robust after transfection compared to the control or vector group in N2A cells. The level of total tau was normalized to the level of DM1A. (B) Quantitative analysis of western blotting data was plotted as graph (n = 4). **P<0.01 compared with the control or vector group.

**Figure 3:**
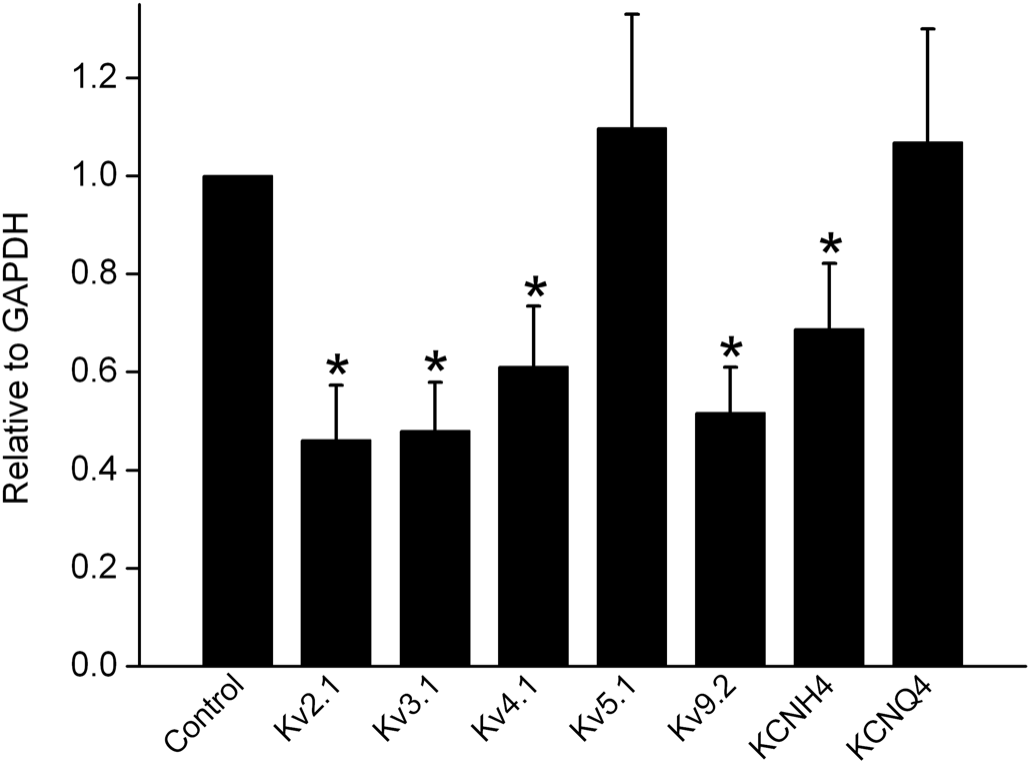
Expressions of Kv channels were reduced by tau transfection. The induction of tau downregulated the mRNA level of Kv2.1, Kv3.1, Kv4.1, Kv9.2, and KCNH4, but no significant changes were found for Kv5.2 and KCNQ4. The statistic analyses of QPCR data were plotted as graph (n = 9). *P<0.05 compared with control.

Although previous studies suggested that both tau and Kv channels were involved in regulation of cellular proliferation [22, 28], little is known about possible interaction of tau with Kv channels. In the experiments here, tau was transiently transfected into N2A cells using Lipofectamine 2000 for 48 h. Total tau was detected by Western blotting with antibody Tau-5 (Fig. 2). In agreement with the previous report [25], a weak signal for endogenous tau expression was found in the control and vector group. Apparently, the robust overexpression of tau after transfection was detectable in N2A cells. Subsequently, the alterations of expression of Kv channels in N2A cells were measured by the performance of QPCR. As shown in Fig. 3, overexpression of tau led to a reduction of expression of several different Kv channels, including Kv2.1, Kv3.1, Kv4.1, Kv9.2, and KCNH4. It should be noted that this treatment barely affected the mRNA levels of Kv5.1 and KCNQ4. The quantitative analyses of these actions indicated that expressions of Kv2.1, Kv3.1, Kv4.1, Kv9.2, and KCNH4, were reduced by 53.9%, 51.9%, 39.0%, 48.3% and 31.3%, respectively. To exclude the effects of empty vector on expression of Kv channels, mock transfections were conducted and no significant alteration was detected by QPCR analysis (data not shown).

**Figure 4:**
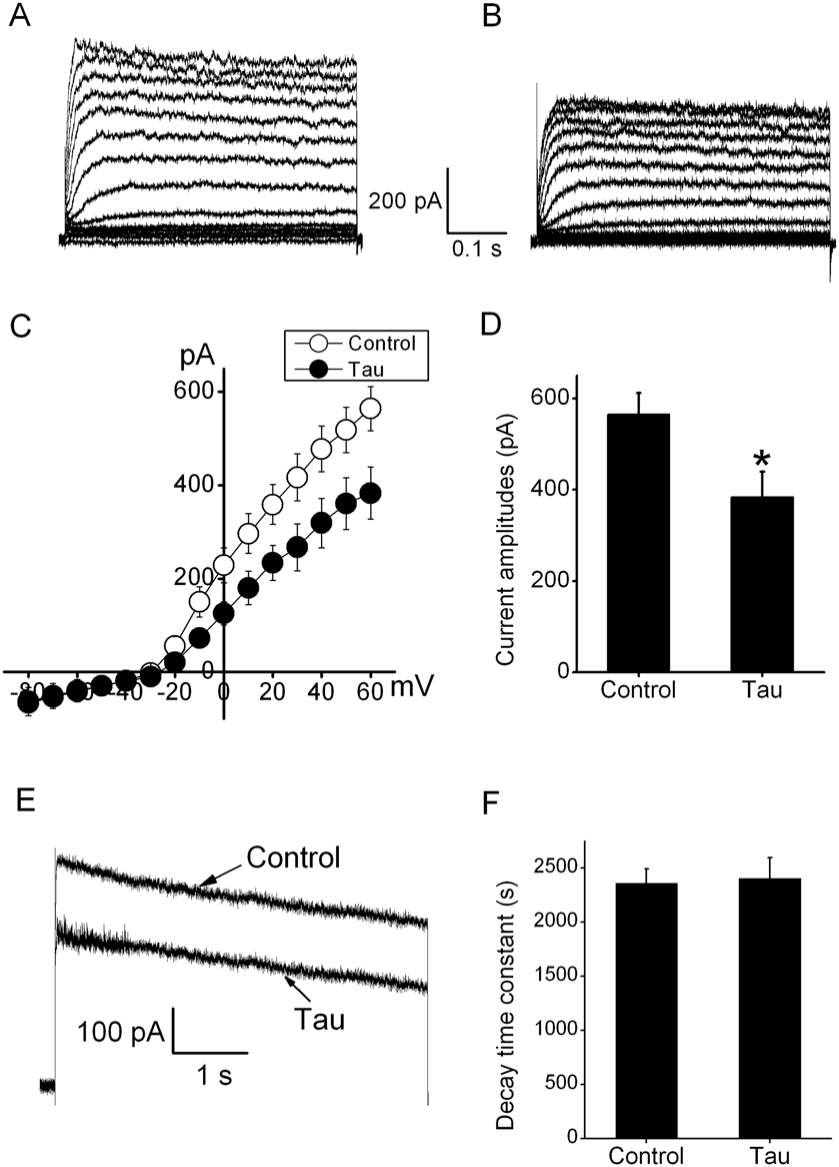
Overexpression of tau inhibited whole-cell currents through Kv channels. (A) The outward Kv currents were evoked by stepped up to +60 mV for 500 ms at a holding potential of -80 mV in N2A cells. (B) These Kv currents were substantially decreased after tau transfection for 48 h. (C) Current–voltage (I-V) relations of outward Kv currents in the control (white circle) and transfection with tau (black circle). (D) The graph showed that statistic analysis of effects of tau overexpression on whole-cell Kv currents (n = 8). *P<0.05 compared with control. (E) Representative current traces in the absence and presence of tau overexpression. (F) The statistic analysis of effects of tau overexpression the decay time constant at +60 mV (n = 9). *P>0.05 compared with control.

As a concomitant of decreased expression of Kv channels, the macroscopic currents through these channels should be declined after tau transfection. At a holding potential of -80 mV, the outward Kv currents in N2A cells were evoked by depolarization to testing potentials ranging from -80 mV to +60 mV (Fig. 4A). After transfection of N2A cells with tau for 48 h, the outward Kv currents appeared to be significantly declined at different testing potentials (Fig. 4B). In addition, the current–voltage curves (I–V), which were constructed by the data of current amplitudes measured at the various testing pulses, clearly showed the inhibitory effects of tau transfection on outward Kv currents (Fig. 4C). At +60 mV, overexpression of tau reduced currents from 564.3 ± 47.5 pA to 383.1 ± 55.8 pA (Fig. 4D). However, this treatment did not affect inward currents, even at -80 mV. To examine the effects of tau overexpression on the decay rate of outward Kv currents, whole-cell currents elicited during 5 s depolarizing voltage step to +60 mV from the holding potential of -80 mV were well fitted with a single exponential function. In the absence and presence of tau overexpression, the decay time constant τ at +60 mV were 2360.1 ± 131.3 ms and 2405.3 ± 192.6 ms, respectively (Fig. 4E, F). These results showed that tau induction unaffected the decay process of Kv currents (Fig. 4F).

### Transfection of tau and blockade of Kv channels improved proliferation rates of N2A cells

**Figure 5:**
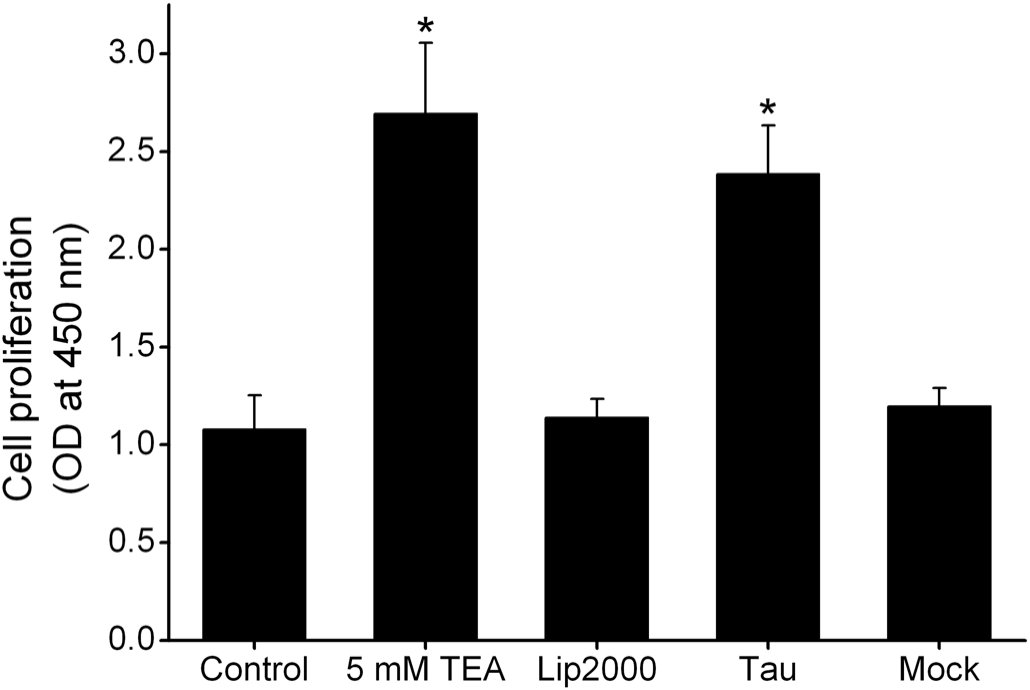
Effects of tau transfection and TEA on cell proliferation. The graph showed that both tau overexpression and TEA (Kv channels blocker) were able to improve the proliferation rate of N2A cells (n = 8). No significant change was detected for treatment with Lipofectamine 2000 or mock transfection. The proliferation rates of each testing group were normalized to control. The * denoted p<0.05 compared with control.

The present study found that overexpression of tau downregulated the expression of Kv channels in N2A cells. It is well known that Kv channels are the important modulators of cell proliferations in distinct tissues [20]. To explore the pathophysiological sense in proliferation for this finding, further experiments were conducted to test the effects of tau overexpression on the proliferation of N2A cells. At 48 h after transfection, cell proliferation was measured by WST-8 assay as described above (see material and methods). N2A cells without any treatment were taken as the control. In contrast to the control, transfection with tau significantly improved the proliferation of N2A cells by 120.9%, but no significant effect was detected after mock transfection (Fig. 5). Moreover, incubation with TEA, a general blocker of Kv channels, increased the proliferation of N2A cells by 149.3% compared with control (Fig. 5). Certainly, overexpression of tau maybe modulated the cell proliferation through one of its candidate target: Kv channels.

### Tau overexpression declined the expression and corresponding currents of Kv2.1 and improved the growth of HEK293 cells

**Figure 6:**
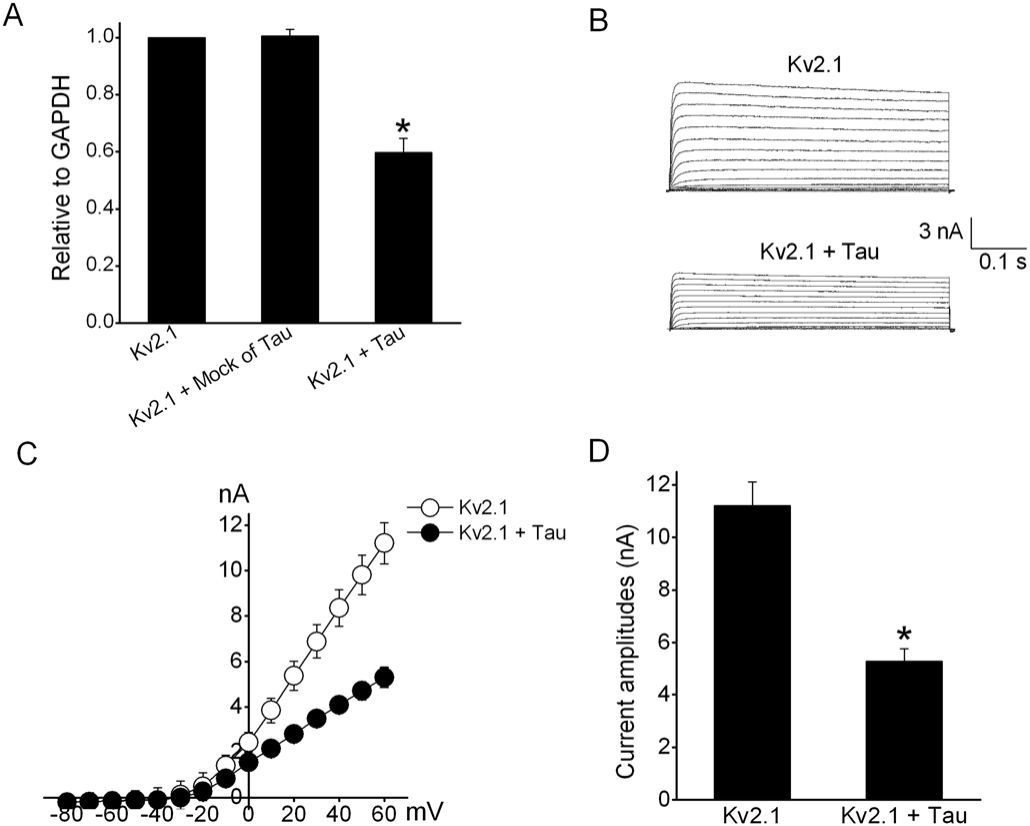
Effects of cotransfection with tau and Kv2.1 in HEK293 cells. (A) QPCR data showed that the induction of tau downregulated the mRNA level of Kv2.1. But no change was produced after the transfection of an empty vector (mock) of tau (n = 8). The * denoted p<0.05 compared with control Kv2.1 (B) The representative current traces of Kv2.1 before and after tau transfection. Currents were evoked by a 500 ms long voltage pulse to potentials from -80 mV to +60 mV at a holding potential of -80 mV. (C) Current–voltage (I-V) relations of Kv2.1 currents in the absence and presence of tau. (D) The statistic analysis of effects of tau transfection on macroscopic Kv2.1 currents in HEK293 cells (n = 8). The * denoted p<0.05 compared with Kv2.1 currents.

**Figure 7:**
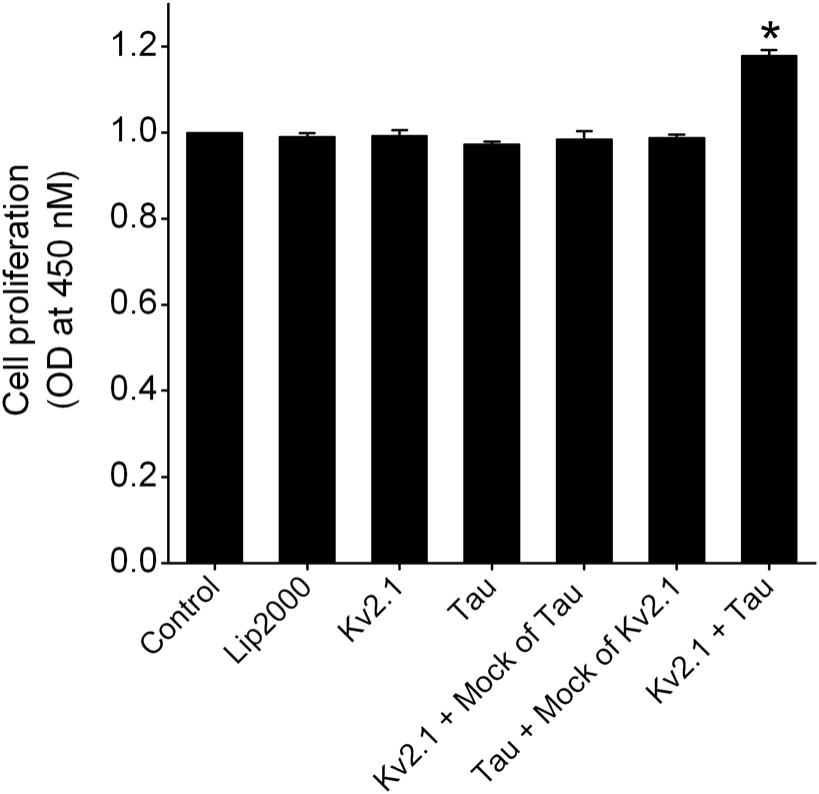
Action of cotransfection with tau and Kv2.1 on the proliferation of HEK293 cells. HEK293 cells were cotransfected with Kv2.1 and tau, Kv2.1 and empty vector (mock) of tau, and tau and empty vector (mock) of Kv2.1, as well as only transfected with Kv2.1 or tau. The proliferation rates of each testing group were normalized to control (n = 7). The * denoted p<0.05 compared with control.

To obtain the direct evidence about the effects of tau overexpression on Kv channels, we conducted the cotransfection of tau and Kv2.1, one of the Kv members with the highest inhibitory percentage following induction of tau in N2A cells (see Fig. 3), into HEK293 cells without the expression of both tau and Kv2.1. At 48 h after cotransfection with tau, the mRNA levels of Kv2.1 were significantly declined by 49.7% compared with single Kv2.1 transfection, whereas no effect was observed for cotransfection with empty vector (mock) of tau (Fig. 6A). Accordingly, whole-cell currents of Kv2.1 in HEK293 cells cotransfected with tau were potentially reduced with respect to the single Kv2.1 transfection. At +60 mV, overexpression of tau declined Kv2.1 currents from 11.2 ± 0.9 nA to 5.7 ± 0.5 nA (Fig. 6D). The representative Kv2.1 current traces and current–voltage (I–V) curves were presented in Fig. 6B and 6C, respectively. We next measured the effects of interaction of tau and Kv2.1 on the growth of HEK293 using a method described above. As shown in Fig. 7, transfection with tau leaded to a weak and significant increase in proliferation of HEK293 cells in comparison to single Kv2.1 transfection, but no alteration was visible after mock transfection.

## Discussions

Kv channels localized to plasma membrane are the primary determinants of action potential repolarization in many kinds of cells such as cardiomyocyte and neuron [19]. Tau proteins, a basic component of intraneuronal inclusions, have essential functions in promoting tubulin polymerization and stabilizing microtubules [4]. So far, there is not any report about the interaction of tau with Kv channels in various tissues. In the current study here, we found that overexpression of tau in N2A cells significantly reduced the mRNA levels of Kv channels, including Kv2.1, Kv3.1, Kv4.1, Kv9.1, and KCNH4. Furthermore, the outward whole-cell Kv currents were substantially attenuated by induction of tau into N2A cells. These data implicated that tau was certainly involved in regulating the expression of Kv channels. However, the underlying mechanisms for the effects of tau on these channels remained unclear. Previous studies indicated that the expressions of various mRNA and protein induced the formation of an RNA-protein complex (RNP), which was considered as post-transcriptional regulatory complexes of gene expression. Atlas et al. reported that IMP-1 (IGF-II mRNA binding protein 1) associates with HuD and G3BP-1 (RAS-GAP SH3 domain binding protein) proteins and binds directly to tau mRNA in P19 neuronal cells [29]. The formation of tau RNP containing the G3BP1 and IMP1 protein could modulate the tau isoform expression and induce neuronal sprouting [30]. In the present study here, the induction of exogenous tau could elevate the level of tau mRNA and caused RNA granule (tau RNP) formation, which might lead to the downregulation of other mRNA populations such as Kv channels. It is interesting to explore the exact causes for this phenomenon in the future.

Our data presented here revealed that transfection with tau could improve the cell growth. Moreover, cell proliferation induced by tau overexpression was at least partially attributed to the decreased expression of Kv channels. These effects were demonstrated in both native N2A cells and the heterologous expression system. Enhanced levels of tau protein induced by estrogen can increase the growth of ER-positive breast cancer tissues by reducing their sensitivity to taxane [3] and paclitaxel [16], which are important chemotherapy drugs for cancer. The prevention of binding of those drugs to the inner surface of microtubules by tau may partly explain that phenomenon. Our results suggested that the modulation of Kv channel expression by tau overexpression also resulted in an increase of cell proliferation. Thus, under conditions of elevated level of tau, both decreased expression of Kv channels and obstruction of binding of chemotherapy drugs to the inner surface of microtubules could contribute to the low sensitivity to those chemotherapy drugs in ER-positive cancer tissues.

Kv channels play crucial roles in various functions of cell such as maintenance of membrane potential, repolarization of the action potential, and regulation of neuronal firing patterns as well as cell proliferation and apoptosis [20, 31, 32]. Numerous studies demonstrated that Kv channels participated in the regulation of the proliferation in many kinds of cell [32]. As mentioned above, tumor cells are more depolarized than healthy cells; membrane depolarization also facilitates the growth of tumor cells [17, 18]. Therefore, inhibition of Kv channels by overexpression of tau may reduce K^+^ efflux, and directly induce depolarization to improve cell growth. Alternatively, published data indicated that cytoplasmic K^+^ efflux facilitated apoptosis [33]. In agreement with this notion, the reduction of K^+^ efflux owing to the higher expression of tau may also attenuate apoptosis and accordingly contribute to the cell growth. Furthermore, previous finding indicated that elevated levels of intracellular Ca^2+^ in response to opening of voltage-gated Ca^2+^ channels (VGCC) favored tumor cell growth [34, 35, 36]. Thus, decreased Kv currents after tau overexpression could lead to a depolarization of membrane potential; subsequently, activation of VGCC could increase the cytoplasmic Ca^2+^ levels to trigger cell cycle.

Collectively, the present study demonstrates for the first time that overexpression of tau inhibited the expression of Kv channels and related macroscopic currents in N2A cells and the heterologous expression system. Both the induction of tau expression and the blockage of Kv channels could improve the proliferation of N2A cells. Our evidence provides an alternative explanation for low sensitivity to anti-cancer chemicals in ER-positive cancer tissues.
